# Passive Reflectance Sensing and Digital Image Analysis Allows for Assessing the Biomass and Nitrogen Status of Wheat in Early and Late Tillering Stages

**DOI:** 10.3389/fpls.2018.01478

**Published:** 2018-10-10

**Authors:** Salah Elsayed, Gero Barmeier, Urs Schmidhalter

**Affiliations:** Department of Plant Sciences, Technical University of Munich, Freising, Germany

**Keywords:** digital agriculture, high-throughput sensing, imaging, nitrogen management, phenomics, precision phenotyping, precision farming, spectrometry

## Abstract

Proximal remote sensing systems depending on spectral reflectance measurements and image analysis can acquire timely information to make real-time management decisions compared to laborious destructive measurements. There is a need to make nitrogen management decisions at early development stages of cereals when the first top-dressing is made. However, there is insufficient information available about the possibility of detecting differences in the biomass or the nitrogen status of cereals at early development stages and even less comparing its relationship to destructively obtained information. The performance of hyperspectral passive reflectance sensing and digital image analysis was tested in a 2-year study to assess the nitrogen uptake and nitrogen concentration, as well as the biomass fresh and dry weight at early and late tillering stages of wheat from BBCH 19 to 30. Wheat plants were subjected to different levels of nitrogen fertilizer applications and differences in biomass, and the nitrogen status was further created by varying the seeding rate. To analyze the spectral and digital imaging data simple linear regression and partial least squares regression (PLSR) models were used. The green pixel digital analysis, spectral reflectance indices and PLSR of spectral reflectance from 400 to 1000 nm were strongly related to the nitrogen uptake and the biomass fresh and dry weights at individual measurements and for the combined dataset at the early crop development stages. Relationships between green pixels, spectral reflectance indices and PLSR with the biomass and nitrogen status parameters reached coefficients of determination up to 0.95^∗∗^ through the individual measurements and the combined data set. Reflectance-based spectral sensing compared to digital image analysis allows detecting differences in the biomass and nitrogen status already at early growth stages in the tillering phase. Spectral reflectance indices are probably more robust and can more easily be applied compared to PLSR models. This might pave the way for more informed management decisions and potentially lead to improved nitrogen fertilizer management at early development stages.

## Introduction

Nitrogen is considered as an essential element for plant development because it is a component of chlorophyll, amino acids, proteins and enzymes (Erisman et al., 2009). Plant metabolism requires an efficient supply of nitrogen, and the addition of nitrogen increases the efficiency of photosynthesis to produce carbohydrates (Peng et al., 2014). Indicators for crop nitrogen requirements are the aboveground biomass, nitrogen concentration, and nitrogen uptake, which were investigated in this study. These variables must be determined spatially and temporally in the field to match the crop requirements as closely as possible (Diacono et al., 2013; Geesing et al., 2014). To optimize the nitrogen fertilizer application regime as well as its timing in crop production it is essential to know the adequate nitrogen content for a certain amount of biomass or the nitrogen uptake (Nguyen and Kant, 2018).

Timely nitrogen management decisions at early development stages are required, but this information is particularly limited during the early crop development, when *N* management assessments are often diagnostic of eventual crop performance and when remedial fertilizer application decisions could be made (Eitel et al., 2014). In many countries, critical nitrogen decisions are made at the beginning of the season. This is the particularly also the case in regions where a shortage of water leads only to modest yields of small grains, and therefore only one nitrogen application at a rather early growth stage is made, e.g., in the United States or Australia. However, this is also the case in continental regions in Europe where due to the occurrence of more frequent drought spells early in the season timely nitrogen applications are preferred. In contrast in other regions such as West-and Central Europe, characterized by fertile soils and a favorable climate allowing for much higher yields, farmers frequently apply nitrogen to winter wheat in three or even four top dressings.

To support timely and early nitrogen application decisions at early crop growth stages the analysis of soil mineral *N* at the beginning of the growth period of arable crops is well established in several countries in Europe but also in the United States corn-belt region. Hence at the start of the vegetation period, the analysis of the soil mineral *N* is widely used as “pre-plant nitrate test” for spring-sown crops like corn (e.g., Fox et al., 1989; Forrestal et al., 2014). In Denmark, soil mineral-*N* analysis provides an “N prognosis” each spring for the whole country, which is divided into three climatic areas and four soil types (Olfs et al., 2005). With such a prognosis being indicative of the regional soil mineral-*N*, the farm- or field-specific soil mineral *N* status, however, remains mostly unknown, since only a low percentage of the fields is regularly analyzed concerning soil mineral *N*.

At the beginning of the vegetation period, therefore, nitrogen applications are most often purely empirically based, and very few rely on simultaneous determinations of the available residual soil nitrogen. Although simplified on-farm soil nitrate tests have been developed (Schmidhalter, 2005) and have been successfully tested in other regions (Yue et al., 2012), their adoption is very limited or remains slow. Hence, there is a need to arrive at other rational and simplified decisions, particularly at early development stages. In contrast in later growth stages, the usage and potential of detecting the nitrogen status of small grain cereals with plant sensors-based diagnostic information for nitrogen recommendations are well established (Samborski et al., 2009). Ample plant growth with enhanced differences in biomass and the nitrogen status at later growing stages lends to a comfortable and non-invasive detection of the biomass and nitrogen status (Mistele and Schmidhalter, 2010; Erdle et al., 2011). However, this is confronted with difficulties at early development stages. The small size of plants creates observational challenges leading to a relatively large ground instantaneous field of view of optical sensors often dominated by soil (Eitel et al., 2014). Hence, this study investigated how precisely differences in plant biomass and the plant nitrogen status could be detected in two seasons, both by destructive and non-destructive methods at early growth stages of wheat plants.

Destructive methods to estimate the *N* content are considered as very accurate but time-consuming and expensive (Erdle et al., 2011). Precision farming and high-throughput phenotyping by using spectral reflectance measurements and digital imaging has the potential to provide more information to make more informed management decisions on a canopy scale in real time (Schmidhalter et al., 2001; Mistele and Schmidhalter, 2008a; Elsayed et al., 2011; Erdle et al., 2011; Kipp et al., 2014a,b). In the past reflectance sensors have widely been used to measure several canopy variables. Spectral measurements can indirectly assess the biomass (Hansen and Schjoerring, 2003; Erdle et al., 2011; Winterhalter et al., 2013; Becker and Schmidhalter, 2017), the nitrogen content (Lukina et al., 1999; Li et al., 2014) and the nitrogen uptake (Mistele and Schmidhalter, 2008b; Winterhalter et al., 2013; Barmeier and Schmidhalter, 2017).

For example, Lukina et al. (1999) found reasonably good relationships between the normalized difference vegetation index (NDVI) and the dry weight and nitrogen uptake compared with the nitrogen concentration under four nitrogen rates at Feekes growth stages four and five corresponding to the pseudostem erection stage or BBCH growth stage 30 (BBCH stages according to the Federal Biological Research Centre for Agriculture and Forestry, 2001). Li et al. (2014) found poor relationships between several spectral indices with the nitrogen concentration at the beginning of the shooting growth stage. Mistele and Schmidhalter (2008b) found that the index R_780_/R_740_ presented an excellent relationship with nitrogen uptake compared with the nitrogen concentration from BBCH 27 to BBCH 71. Erdle et al. (2011) indicated that the near infrared-based index R_760_/R_730_ revealed to be the most potent and temporarily stable index indicating the nitrogen status of wheat from BBCH 30 to BBCH 69. It is therefore conceivable that particularly at later growth stages, biomass as well as the nitrogen status can successfully be detected and are amenable to informed management decisions. However, there is a lack of detailed studies indicating the possibility of detecting non-destructively differences in plant growth and the nitrogen status at early development stages such as tillering and only very few studies exist where both destructive reference measurements and non-destructive spectral or image-based measurements were compared. Biomass sampling is confronted with difficulties at early development stages, requiring tedious manual samplings (Kipp et al., 2014a) and requires extreme care to avoid artifacts because of differences in the cutting height, and adhering soil particles need to be carefully removed. Therefore, simplified but accurate procedures have to be developed to avoid biased measurements. So far most comparisons were based on indirect comparisons, such as digital versus reflectance measurements, or using other methods such as the line intersection method (Phillips et al., 2004; Scotford and Miller, 2005; Booth et al., 2006; Kipp et al., 2014a). To test the feasibility of both destructive and non-destructive techniques a sufficient range in biomass density and nitrogen uptake is required. This can be achieved by varying the seeding rate and the nitrogen fertilizer application.

Using digital cameras and image processing techniques is less expensive than other techniques, such as terrestrial active and passive reflectance sensing or satellite imagery. The applications of digital cameras by using color images has been proven to be a potential source to estimate canopy variables such as crop growth (Paruelo et al., 2000; Lee and Lee, 2013), leaf chlorophyll content (Hu et al., 2013), nitrogen status (Li et al., 2010; Jia et al., 2014; Baresel et al., 2017), differentiation of early plant vigor (Kipp et al., 2014a), senescence of wheat (Adamsen et al., 1999) and vegetation cover (Lukina et al., 1999). For example, Paruelo et al. (2000) found that the percentage of green pixels of images and green grass biomass showed a good curvilinear relationship when data were pooled from three sampling dates. A strong relationship was found between the relative amount of green pixels, and the early vigor index of 50 winter wheat cultivars at BBCH 23 and 28 (Kipp et al., 2014a). In direct agreement with the purpose of this work Flowers et al. (2001) demonstrated that tiller density at BBCH 25 was consistently correlated (0.67 ≤*r* ≤ 0.87) with NIR digital counts obtained from aerial imaging across four sites. Including the within-field tiller density allowed to obtain a high correlation (*r* = 0.88) between relative tiller density and relative NIR digital counts across environments.

Spectral assessments of the biomass and nitrogen status at early wheat growth stages were most frequently done not before Feekes 4–5, corresponding to the BBCH growth stage 30, or pseudostem erection, by using active sensors in the United States. The same holds true for the use of either active or passive reflectance sensors which are generally recommended for the second top dressing at around the BBCH growth stages 30–32 in West Europe. It has been reported that with small grains passive reflectance sensing is seldom used for the first dressing (Link et al., 2004; Heege et al., 2008) and applications are mostly limited to the second and later dressings. Whether an indication of the biomass and chlorophyll content could be obtained at the time of the first dressing remains to be shown (Heege et al., 2008).

The purpose of this work was therefore to evaluate the performance of hyperspectral passive reflectance and digital imaging as proximal sensing techniques for assessing the biomass and nitrogen status of wheat at early growth stages from BBCH 19 to 30 in 2 years.

## Materials and Methods

### Field Experiments and Design

Field experiments were conducted at the Dürnast research station of the Chair of Plant Nutrition belonging to the Technical University of Munich in southwestern Germany (11°41′60″ E, 48°23′60″N). Dürnast is characterized by a sub-oceanic climate, with mild, cloudy winters and warm summers. The experimental design in 2014 was a split-plot with seeding density as the main plot and nitrogen treatments as subplots with eight replications totaling 64 plots. The experimental design consisted of two seeding rates, a standard sowing rate of 250 kernels, a double rate applied as cross-sowing of 500 kernels per square meter, and four fertilizer rates with 0, 100, 160, and 220 kg nitrogen per hectare. The cross-seeding imitated a frequent observation of double-seeding rates, which are partly unavoidable at the headlands of fields. In 2014 the fertilizer was applied at three different growth stages at BBCH 15, BBCH 32, and 49 as an ammonia nitrate urea solution (AHL). Additionally, 30 kg nitrogen per hectare was previously applied to half of the plots in late autumn in 2013 to create a more considerable variability in biomass and the nitrogen status. The wheat cultivar Kerubino was sown on 15 October 2013 on calcaric Cambisol consisting of silty loam. The plots comprised 14 rows spaced 12.5 cm apart and had a length of 6 m each. Herbicide and fungicide treatments were applied in all trials when necessary.

To validate the experimental findings obtained in 2014 a subsequent independent verification trial was conducted on a nearby conventionally cultivated farm site in 2016 with winter wheat. Thirty areas of interest were selected either on the headlands of the field, in a zone with overlapping seeding beds reflecting a higher seeding density, or in the middle of the field with an average seeding rate. The wheat cultivar Kerubino was sown on 25 October 2015 with a seeding rate of 300 kernels per square meter. Due to less favorable weather conditions in autumn 2015, the germination rate was slightly lower compared to the field trial in 2014. The experiment was fertilized with 80 kg nitrogen per hectare, and the fertilizer was applied at BBCH 20 as ammonium sulfate nitrate. Pesticide treatments followed local recommendations.

### Biomass Sampling

Biomass sampling was performed four times, on 4 March 2014 (BBCH 19), 17 March 2014 (BBCH 22), 1 April 2014 (BBCH 25), and 14 April 2014 (BBCH 30) (BBCH stages according to the Federal Biological Research Centre for Agriculture and Forestry, 2001), and two times in 2016 on 29 March (BBCH 19) and 4 April (BBCH 22). To determine fresh biomass the aboveground biomass and their roots were removed from a 0.25 m^2^ area. To eliminate biases in cutting height the plants were removed with their adhering roots, and the latter were cut away in the subsequent procedure. Fresh biomass was put into plastic bags, immediately washed and dried in a salad spinner, and the fresh weight was determined. This step was necessary to remove adhering soil particles from the plants. Subsequently, samples were dried at 65°C until there was no change in dry weight. The dried plant samples were finely ground, and the *N* content (g N g^-1^ dry weight) was determined using an isotope ratio mass spectrometer with an ANCA SL 20–20 preparation unit (Europe Scientific, Crewe, United Kingdom). The aboveground *N* uptake (kg ha^-1^) was calculated as plant dry weight multiplied by the total *N* content.

### Digital Image Analysis

Images of plots were taken before biomass sampling at precisely the same position at four dates using a Nikon D5100 reflex camera with a pixel resolution of 3696 × 2448. The camera was manually held and oriented vertically downward to the canopy at the height of approx. One hundred and fifty centimeter under overcast conditions to avoid shadows and unwanted reflections. To have a comparable area photographed a frame (50 × 50 cm) was used and afterward the area of interest was cut out before image analysis. The digital images were processed by using the Java-based open-source software ImageJ 1.49p, U.S. National Institutes of Health, Bethesda, MD, United States. To analyze the images according to their color spectra the RGB images were converted to the HSB color space (Hue, Saturation, and Brightness). The related color threshold values to segment green pixels from the rest are shown in **Table 1**. After this thresholding method a binary image was created, that is segmented into green pixels (plant material) and residual colors (mostly soil and dead plant material). The relative amount of green pixels was calculated by:

$$\text{Green pixels in \%=}\frac{\text{Amount of green pixels}}{\text{Total amount of pixels}}*100$$

**Table 1:** Color threshold values used for processing green pixels.

Threshold level	Hue	Saturation	Brightness
Lower threshold level	45	0	0
Upper threshold level	120	255	255


### Spectral Reflectance Measurements

The up- and downwelling radiation was detected by a reflectance sensor HandySpec Field^®^ (tec5, Oberursel, Germany) measuring at wavelengths between 302 and 1148 nm with a bandwidth of 2 nm. The sensor was connected to a portable computer and geographical positioning system (GPS), Qstarz BT-Q818XT (Qstarz International Co., Ltd., Taiwan, Republic of China). The passive reflectance sensor consists of two units: One unit is linked to a diffuser and measures the light radiation as a reference signal, the second unit measures the canopy reflectance with a fiber optic simultaneously (Mistele and Schmidhalter, 2008b; Elsayed et al., 2011). The aperture of the optics is set at 12°, and at 1-m height, the field of view was 0.2 m^2^. The sensor outputs are co-recorded along with the GPS coordinates, and for each position, the actual sensor output is co-referenced and recorded with a self-written software. Readings within one plot are averaged to single values per plot. The canopy reflectance was calculated and corrected with a calibration factor obtained from a reference standard. Spectral measurements were mostly taken on sunny days at the nadir direction about 1 m above the canopy. Spectral reflectance measurements were done on all plots or sites in 2014, on March 4 (BBCH 19), March 17 (BBCH 22), April 1 (BBCH 25), and April 14 (BBCH 30 growth stage) and in 2016 on March 29 (BBCH 19) and April 4 (BBCH 22). Sensor measurements and biomass samplings were done on the same days.

### Spectral Reflectance Indices

In **Table 2** 12 different spectral indices are listed with references that were used to evaluate the relation to the biomass and the nitrogen status. Known and novel indices were tested. Using a contour map analysis for all wavelengths of the passive hyperspectral sensor (from 302 to 1048 nm) optimized normalized difference indices could be identified, which generally presented more stable and strong relationships with the biomass fresh and dry weight, the nitrogen concentration and the nitrogen uptake. Contour maps are matrices of the coefficients of determination of the biomass fresh and dry weights and the nitrogen concentration and nitrogen uptake with all possible combinations of binary, normalized spectral indices. The R package “lattice” from the software R statistics version 3.0.2 (R foundation for statistical computing 2013) was used to produce the contour maps from the reflectance readings, and 19 wavelengths (550, 572, 590, 624, 640, 656, 660, 670, 710, 720, 730, 740, 760, 774, 780, 800, 820, 850, and 952 nm) were selected to calculate optimized reflectance indices (**Table 2**).

**Table 2:** Spectral reflectance indices, formula and references of different previously developed and new spectral indices developed and used in this study.

Spectral reflectance indices	Formula	Reference
Normalized difference of reflectance between 590 - 550 and 590 + 550 nm	(R_590 -_ R_550_)/(R_590_ + R_550_)	This work
Normalized difference of reflectance between 624 - 572 and 624 + 572 nm	(R_624 -_ R_572_)/(R_624_ + R_672_)	This work
Normalized difference of reflectance between 710 - 640 and 710 + 640 nm	(R_710 -_ R_640_)/(R_710_ + R_640_)	This work
Normalized difference of reflectance between 760 - 550 and 760 + 550 nm	(R_760 -_ R_550_)/(R_760_ + R_550_)	Hackl et al., 2012
Normalized difference of reflectance between 760 - 730 and 760 + 730 nm	(R_760 -_ R_730_)/(R_760_ + R_730_)	Barnes et al., 2000
Normalized difference vegetation index (NDVI)	(R_780 -_ R_670_)/(R_780_ + R_670_)	Rouse et al., 1974
Normalized difference of reflectance between 780 - 720 and 780 + 720 nm	(R_780 -_ R_720_)/(R_780_ + R_720_)	This work
Normalized difference of reflectance between 780 - 740 and 780 + 740	(R_780 -_ R_740_)/(R_780_ + R_740_)	Adapted from Mistele and Schmidhalter, 2008a
Normalized difference of reflectance between 800 - 720 and 800 + 720 nm	(R_800 -_ R_720_)/(R_800_ + R_720_)	This work
Normalized difference of reflectance between 820 - 660 and 820 + 660 nm	(R_820 -_ R_660_)/(R_820_ + R_660_)	This work
Normalized difference of reflectance between 850 - 730 and 850 + 730 nm	(R_850 -_ R_730_)/(R_850_ + R_730_)	This work
Normalized difference of reflectance between 952 - 720 and 952 + 720 nm	(R_952 -_ R_720_)/(R_952_ + R_720_)	This work


### Statistical Analysis

For the statistical analysis Sigmaplot for Windows v.12 (Systat Software Inc., Chicago), and SPSS 16 (SPSS Inc., Chicago, IL, United States) was used. To analyze the relationship between the spectral indices listed in **Table 2** and the biomass fresh and dry weight, as well as the nitrogen concentration and nitrogen uptake, simple linear regressions were calculated. Coefficients of determination and significance levels were determined by using nominal alpha values of 0.05, 0.01, and 0.001.

To calibrate and validate partial least square models the Unscrambler X multivariate data analysis software version 10.2 (CAMO Software AS, Oslo, Norway) was used. Single wavebands derived from the same spectra usually contain redundant information (Sharabian et al., 2014). To cope with redundancy in the input variables Partial Least Square Regression (PLSR) creates orthogonal latent variables across the input variables (single wavebands) and relates them to the target variables (the biomass fresh and dry weights, the nitrogen concentration and nitrogen uptake). PLSR searches the sensitive information from spectral reflectance for all wavelengths. All wavelengths from 400 to 1000 were used as input variables in the PLSR models for the model datasets for the 2 years. For determining the model quality, a seven-fold cross-validation approach was applied for the PLSR analysis. Calibration and validation quality of the models is presented through adjusted coefficients of determination of calibration (*R*^2^ cal) and validation (*R*^2^ val), root mean square errors for calibration (RMSEC) and validation (RMSEV), and the slope of the linear regressions between observed and predicted values of the biomass fresh and dry weights, the nitrogen concentration and nitrogen uptake, is shown.

## Results

### Variation in the Green Pixel Percentage, Biomass Fresh and Dry Weight, Nitrogen Concentration and Nitrogen Uptake at Varied Nitrogen Fertilizer Application and Seeding Rates

The minimum, maximum and mean values of the destructively measured values are shown for each parameter of 64 plots at four levels of nitrogen fertilizer application and two seeding rates in 2014 and 30 plots in 2016 with varying seeding rates in different growth stages in **Tables 3**, **4**. Significant differences (*P* ≤ 0.05) were found for the green pixel percentage, biomass fresh and dry weights, nitrogen concentration and nitrogen uptake among different stages of development. The highest mean values of the green pixel percentage, biomass fresh and dry weights, nitrogen uptake (61.9%, 9187 kg ha^-1^ and 2206 kg ha^-1^, 44.8 kg ha^-1^, respectively) were recorded at BBCH 30 in 2014. In contrast, in 2016 the highest mean value of the nitrogen concentration (5.4%) was recorded at BBCH 22.

**Table 3:** Minimum, maximum and mean values for the green pixel percentage obtained from the image analysis, as well as the destructively obtained parameters nitrogen uptake (kg ha^-1^), nitrogen concentration (%), biomass fresh and dry weight (kg ha^-1^) of wheat at the BBCH stages 19, 22, 25, and 30 in 2014.

Dates	BBCH		Green pixels (%)	Nitrogen uptake (kg ha^-1^)	Nitrogen concentration (%)	Biomass fresh weight (kg ha^-1^)	Biomass dry weight (kg ha^-1^)
04.03.2014	19	Min	8.3	6.8	2.2	968.0	232.0
		Max	35.8	29.2	4.9	3704.0	972.0
		Mean	18.8d	14.4d	3.2a	1913.6d	450.4d
17.03.2014	22	Min	24.5	8.0	1.9	1452.0	340.0
		Max	63.9	34.8	3.6	4812.0	1452.0
		Mean	42.8c	19.6c	2.6b	2952.4c	758.4c
01.04.2014	25	Min	23.0	10.4	1.5	1900.0	472.0
		Max	83.2	58.0	3.3	10324.0	2340.0
		Mean	53.6b	29.2b	2.4c	5306.4b	1218.8b
14.04.2014	30	Min	18.2	10.0	1.3	2552.0	632.0
		Max	87.8	94.0	3.1	15454.0	4304.0
		Mean	61.9a	44.8a	2.0d	9186.8a	2206.0a


*Values with the same letter are not statistically different (*P* ≥ 0.05) between growth stages for each parameter according to Duncan’s test.*

**Table 4:** Minimum, maximum and mean values for the green pixel percentage obtained from the image analysis, as well as the destructively obtained parameters nitrogen uptake (kg ha^-1^), nitrogen concentration (%), biomass fresh and dry weight (kg ha^-1^) of wheat at the BBCH stages 19 and 22 in 2014.

Dates	BBCH		Green pixels (%)	Nitrogen uptake (kg ha^-1^)	Nitrogen concentration (%)	Biomass dry weight (kg ha^-1^)
29.03.2016	19	Min	10.9	8.1	2.6	232.8
		Max	29.3	17.0	3.8	573.2
		Mean	18.8a	12.0a	3.1a	385.5a
04.04.2016	22	Min	31.9	18.1	4.4	365.2
		Max	60.2	41.1	5.4	826.0
		Mean	46.4b	27.3b	5.0b	548.1b


*Values with the same letter are not statistically different (*P* ≥ 0.05) between growth stages for each parameter according to Student’s t-test.*

### The Relationship Between the Green Pixel Percentages Derived From the Image Analysis and the Destructively Analyzed Parameters Biomass Fresh and Dry Weights, Nitrogen Concentration and Uptake

**FIGURE 1:**
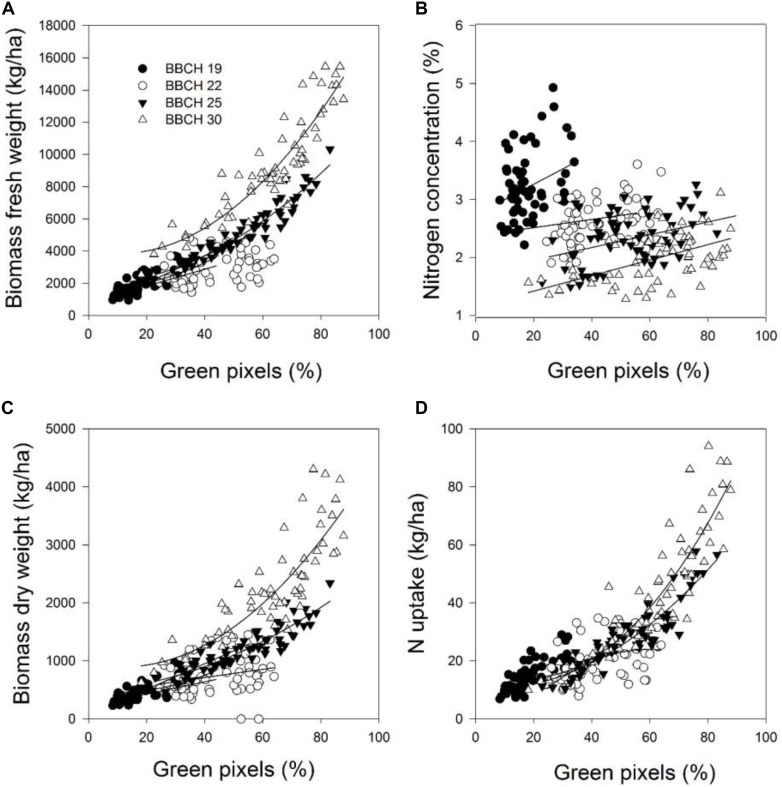
The relationship between green pixels percentage obtained from image analysis and the destructively obtained parameters **(A)** biomass fresh weight, **(B)** biomass dry weight, **(C)** nitrogen concentration, and **(D)** nitrogen uptake of wheat at four growth stages in 2014. Statistical information is given in **Table 5**.

**FIGURE 2:**
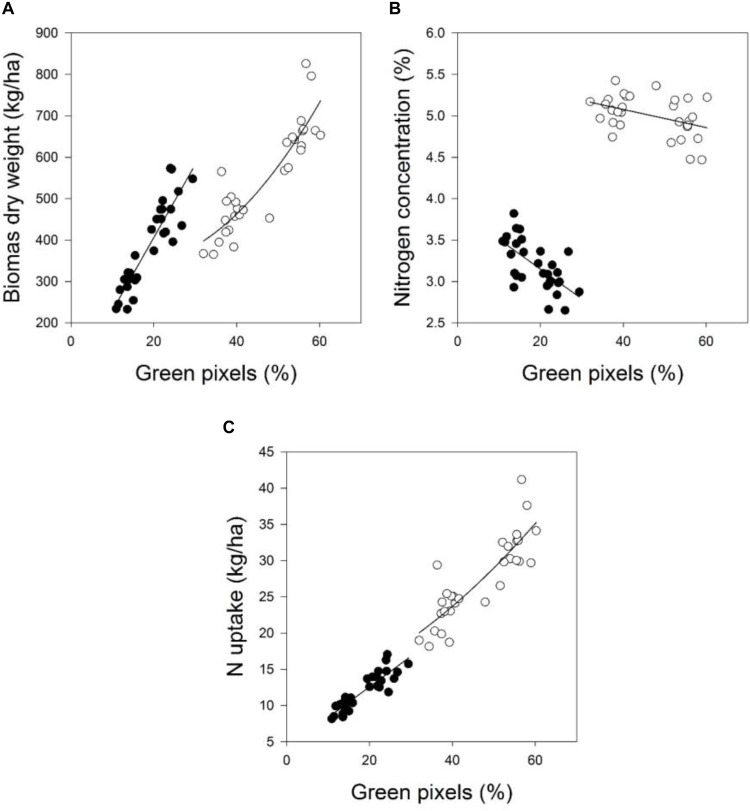
The relationship between green pixels percentage obtained from image analysis and the destructively obtained parameters **(A)** biomass dry weight, **(B)** nitrogen concentration, and **(C)** N uptake of wheat at four growth stages in 2016. Statistical information is given in **Table 6**.

The coefficients of determination for the relationships between the green pixel percentage and the four plant parameters recorded at the individual measurement dates and a combination of the data for each parameter are given in **Tables 5**, **6**. The results demonstrated that the green pixel percentage was significantly related to the four growth parameters (*R*^2^ = 0.06^∗^–0.92^∗∗∗^) at the individual sampling dates and across all sampling dates in the considered years. Stronger relationships between the green pixel percentage and the biomass fresh and dry weight and the nitrogen uptake were found compared with the nitrogen concentration (**Tables 3**, **4** and **Figures 1**, **2**). In 2014, higher coefficients of determination for the biomass fresh and dry weight and the nitrogen uptake with the green pixel percentage were found at BBCH 22, 25, 30 compared with BBCH 19 (**Figure 1**). Linear relationships between the green pixel percentage and the biomass fresh and dry weight and the nitrogen uptake were observed up to a percentage of about 45% green pixels, whereas curvilinear relationships were observed at higher percentages.

**Table 5:** Coefficients of determination (*R*^2^) of relationships obtained from simple regression analysis of the green pixel percentage obtained from the image analysis with the destructively obtained parameters nitrogen uptake, nitrogen concentration, biomass fresh and dry weight of wheat at individual measurements and for the combined measurements.

Dates	BBCH	Nitrogen uptake (kg ha^-1^)	Nitrogen concentration (%)	Biomass fresh weight (kg ha^-1^)	Biomass dry weight (kg ha^-1^)
04.03.2014	19	0.61^∗∗∗^	0.10^∗∗^	0.56^∗∗∗^	0.48^∗∗∗^
17.03.2014	22	0.85^∗∗∗^	0.06^∗^	0.88^∗∗∗^	0.71^∗∗∗^
01.04.2014	25	0.81^∗∗∗^	0.19^∗∗∗^	0.89^∗∗∗^	0.81^∗∗∗^
14.04.2014	30	0.75^∗∗∗^	0.30^∗∗∗^	0.81^∗∗∗^	0.68^∗∗∗^
All dates		0.84^∗∗∗^	0.15^∗∗∗^	0.83^∗∗∗^	0.82^∗∗∗^


*^∗^, ^∗∗^, ^∗∗∗^ Statistically significant at *P* ≤ 0.05, *P* ≤ 0.01, and P ≤ 0.001, respectively.*

**Table 6:** Coefficients of determination (*R*^2^) of relationships obtained from simple regression analysis of the green pixel percentage obtained from the image analysis with the destructively obtained parameters nitrogen uptake, nitrogen concentration, biomass fresh and dry weight of wheat at individual measurements and for the combined measurements.

Dates	BBCH	Nitrogen uptake (kg ha^-1^)	Nitrogen concentration (%)	Biomass dry weight (kg ha^-1^)
29.03.2016	19	0.79^∗∗∗^	0.40^∗∗∗^	0.81^∗∗∗^
04.04.2016	22	0.73^∗∗∗^	0.16^∗∗^	0.77^∗∗∗^
All dates		0.92^∗∗∗^	0.60^∗∗∗^	0.72^∗∗∗^


*^∗^, ^∗∗^, ^∗∗∗^ Statistically significant at *P* ≤ 0.05, *P* ≤ 0.01, and *P* ≤ 0.001, respectively.*

### Contour Map Analysis of the Reflectance Data

**FIGURE 3:**
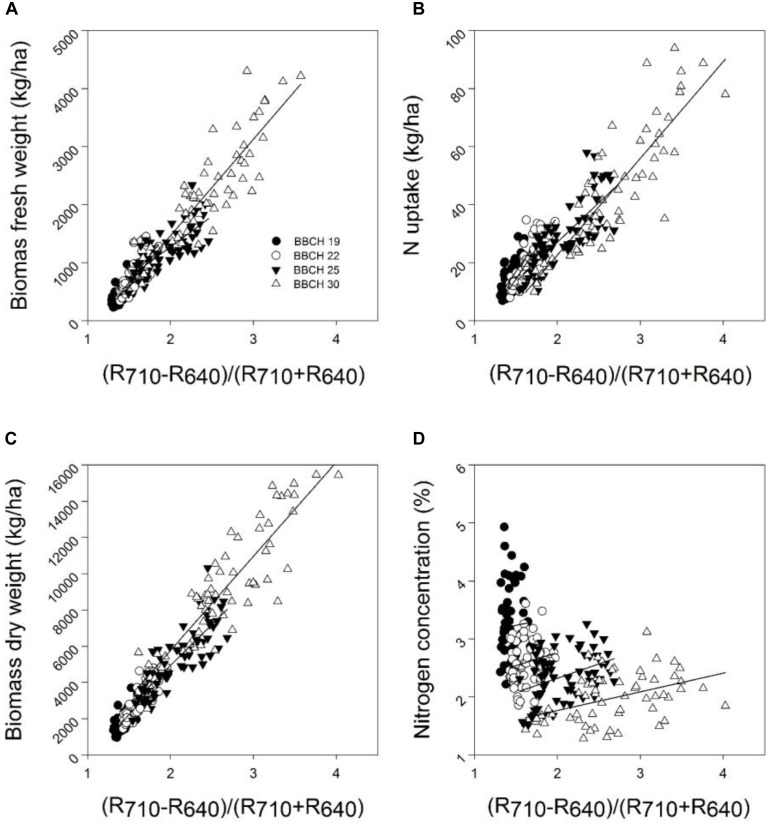
The relationship between the spectral index (R_710_ – R_640_)/(R_710_ + R_640_) and the **(A)** biomass fresh weight, **(B)** biomass dry weight, **(C)** nitrogen concentration, and **(D)** nitrogen uptake of the wheat cultivar Kerubino at four growth stages grown in experimental plots in 2014. Statistical information is given in **Table 7**.

**FIGURE 4:**
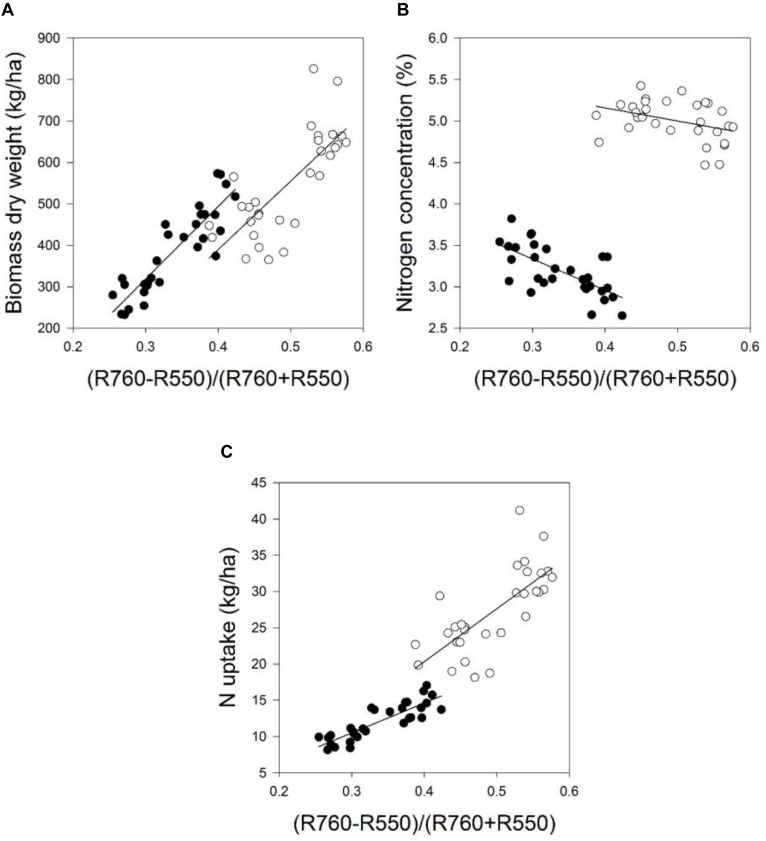
The relationship between the spectral index (R_710_ – R_640_)/(R_710_ + R_640_) and the **(A)** biomass dry weight, **(B)** nitrogen concentration, and **(C)** nitrogen uptake of the field grown wheat cultivar Kerubino at two growth stages in 2016. Statistical information is given in **Table 8**.

Mean coefficients of determination (*R*^2^) of four sampling times for all dual wavelength combinations as a normalized difference index were obtained from the contour map analysis. The contours of the matrices of the passive hyperspectral sensor showed generally distinct relationships with biomass fresh and dry weights and the nitrogen uptake in the visible and near-infrared areas and a combination of them. Significant relationships between the biomass fresh and dry weights and the nitrogen concentration and the nitrogen uptake could be shown for all indices at all measurements (**Tables 7**, **8**). However, the relationship varied considerably amongst the investigated indices (*R*^2^ = 0.11^∗^–0.93^∗∗∗^), as indicated in **Tables 7**, **8**. Indices revealing the closest relationships are illustrated in **Figures 3**, **4** for the years 2014 and 2016, respectively.

**Table 7:** Coefficients of determination of relationships obtained from simple regression analysis of destructively obtained parameters nitrogen uptake, nitrogen concentration, biomass fresh and dry weight with spectral indices (calculated as normalized difference indices) of wheat for each individual measurement date and for all dates (March 4, March 17, April 1, April 14) in 2014 together.

Spectral indices	Nitrogen uptake (kg ha^-1^)					Nitrogen concentration (%)					Biomass fresh weight (kg ha^-1^)					Biomass dry weight (kg ha^-1^)				
				
	4.03	17.03	1.04	14.04	All	4.03	17.03	1.04	14.04	All	4.03	17.03	1.04	14.04	All	4.03	17.03	1.04	14.04	All
590_550	0.64	0.84	0.71	0.73	0.84	0.15	0.11	0.25	0.30	0.19	0.63	0.80	0.74	0.78	0.93	0.53	0.68	0.62	0.76	0.89
624_572	0.66	0.84	0.70	0.72	0.78	0.18	0.11	0.25	0.28	0.18	0.61	0.80	0.74	0.78	0.92	0.50	0.68	0.62	0.76	0.89
710_640	0.46	0.73	0.64	0.78	0.82	0.02	0.04	0.17	0.20	0.18	0.56	0.75	0.73	0.79	0.92	0.52	0.66	0.62	0.80	0.90
760_550	0.45	0.79	0.73	0.82	0.85	0.02	0.06	0.27	0.31	0.17	0.55	0.79	0.75	0.79	0.90	0.51	0.68	0.62	0.75	0.87
760_730	0.63	0.85	0.75	0.80	0.87	0.12	0.14	0.33	0.36	0.10	0.62	0.79	0.72	0.75	0.85	0.53	0.65	0.59	0.68	0.82
780_720	0.61	0.84	0.75	0.80	0.87	0.11	0.13	0.31	0.35	0.10	0.62	0.79	0.72	0.76	0.86	0.53	0.65	0.60	0.69	0.83
780_740	0.63	0.83	0.76	0.79	0.86	0.14	0.15	0.34	0.36	0.09	0.60	0.77	0.70	0.74	0.82	0.50	0.63	0.58	0.67	0.80
780_670	0.53	0.76	0.67	0.76	0.83	0.05	0.07	0.20	0.21	0.11	0.60	0.76	0.72	0.79	0.90	0.53	0.65	0.61	0.77	0.87
800_720	0.62	0.84	0.76	0.81	0.87	0.11	0.13	0.34	0.37	0.12	0.62	0.80	0.72	0.75	0.86	0.53	0.66	0.59	0.70	0.83
820_660	0.55	0.82	0.72	0.81	0.84	0.06	0.08	0.27	0.31	0.19	0.61	0.81	0.75	0.78	0.91	0.55	0.69	0.62	0.75	0.88
850_730	0.60	0.82	0.74	0.80	0.86	0.10	0.14	0.33	0.36	0.11	0.60	0.76	0.70	0.75	0.85	0.51	0.62	0.57	0.69	0.82
952_720	0.59	0.82	0.74	0.79	0.86	0.10	0.13	0.32	0.35	0.10	0.60	0.77	0.70	0.74	0.84	0.52	0.63	0.58	0.68	0.81


*Statistically significant at *R*^*2*^ from 0.09 to 0.12, *P* ≤ 0.05; *R*^*2*^ from 0.13 to 0.18, *P* ≤ 0.01; *R*^*2*^ ≥ 0.19, *P* ≤ 0.001, respectively.*

**Table 8:** Coefficients of determination of relationships obtained from simple regression analysis of destructively obtained parameters nitrogen uptake, nitrogen concentration, biomass fresh and dry weight with spectral indices (calculated as normalized difference indices) of wheat for each individual measurement date and for all dates (March 29, April 4) in 2016 together.

Spectral indices	Nitrogen uptake (kg ha^-1^)			Nitrogen concentration (%)			Biomass dry weight (kg ha^-1^)		
			
	29.03	4.04	All	29.03	4.04	All	29.03	4.04	All
590_550	0.68	0.51	0.83	0.41	0.12	0.49	0.73	0.55	0.69
624_572	0.70	0.51	0.85	0.37	0.12	0.54	0.72	0.55	0.66
710_640	0.74	0.53	0.82	0.47	0.13	0.44	0.81	0.58	0.75
760_550	0.75	0.52	0.84	0.45	0.14	0.50	0.79	0.57	0.73
760_730	0.70	0.49	0.87	0.36	0.15	0.62	0.71	0.55	0.66
780_720	0.70	0.48	0.86	0.37	0.14	0.59	0.72	0.53	0.67
780_740	0.64	0.46	0.86	0.30	0.17	0.64	0.64	0.53	0.64
780_670	0.73	0.51	0.85	0.44	0.12	0.53	0.78	0.55	0.71
800_720	0.70	0.48	0.86	0.37	0.14	0.60	0.71	0.53	0.67
820_660	0.73	0.50	0.85	0.44	0.12	0.52	0.77	0.54	0.71
850_730	0.65	0.44	0.85	0.33	0.15	0.61	0.66	0.50	0.65
952_720	0.64	0.42	0.84	0.30	0.13	0.62	0.63	0.47	0.63


*Statistically significant at *R*^*2*^ from 0.09 to 0.12, *P* ≤ 0.05; *R*^*2*^ from 0.13 to 0.18, *P* ≤ 0.01; *R*^*2*^ ≥ 0.19, *P* ≤ 0.001, respectively.*

### Prediction of Biomass Fresh and Dry Weight, Nitrogen Concentration and Nitrogen Uptake by Partial Least Squares Regression Analysis

**FIGURE 5:**
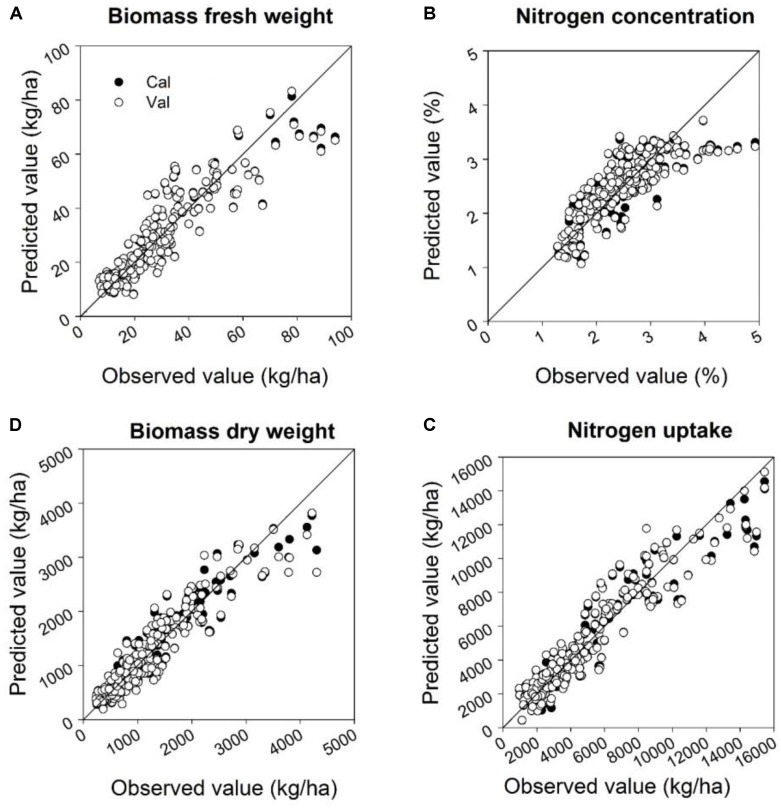
Relationships between the observed and predicted: **(A)** biomass fresh weight, **(B)** biomass dry weight, **(C)** nitrogen concentration, and **(D)** nitrogen uptake at four sampling times for calibration and validation datasets in 2014 using a partial least squares model. For the PLS model a cross-validation was performed. Linear calibration models of all datasets for each parameter were used to validate the model at four measurements dates. Statistical information is given in **Table 9**.

**FIGURE 6:**
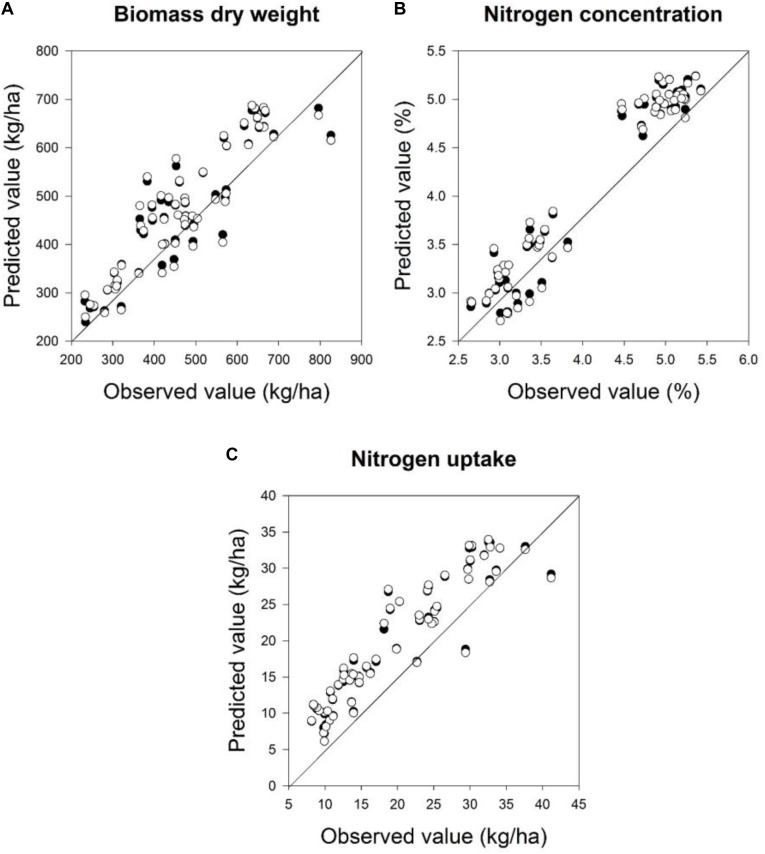
Relationships between the observed and predicted: **(A)** biomass dry weight, **(B)** nitrogen concentration, and **(C)** nitrogen uptake at two sampling times at BBCH 19 and 22 for calibration and validation datasets in 2016 using a partial least squares model. For the PLS model a cross-validation was performed. Linear calibration models of all datasets for each parameter were used to validate the model at four measurements dates. Statistical information is given in **Table 10**.

The quality of the PLSR models is presented through adjusted coefficients of determination of calibration (*R*^2^ cal) and validation (*R*^2^ val), root mean square errors (RMSE cal and val) and the slope of linear regression for calibration and validation models at four measurement dates (**Tables 9**, **10** and **Figures 5**, **6**). The coefficients of determination varied in the 2 years from 0.40^∗∗^ to 0.95^∗∗∗^ for all parameters across all validation data set formations. The coefficients of determination varied from 0.21^∗∗^ to 0.93^∗∗∗^ for all parameters across all validation data set formations, whereas the RMSE varied from 47.5 to 367.4 kg ha^-1^ for the biomass fresh weight, from 26 to 111.2 kg ha^-1^ for the biomass dry weight, from 0.10 to 0.53% for the nitrogen concentration and from 0.8 to 4.2 kg ha^-1^ for the nitrogen uptake across all calibration and validation data set formations. Across all dates of biomass fresh weight samplings, the highest slopes of the calibration and validation data sets were found to be 0.93 and 0.92, respectively (**Tables 9**, **10**).

**Table 9:** Results of calibration (*R*^2^ cal, RMSEC and slope cal), and seven-fold cross-validation (*R*^2^ val, RMSEV and slope val) partial least square regression models of the relationship between spectral reflectance from 400 to 1000 nm and biomass fresh weight, biomass dry weight, nitrogen uptake and nitrogen concentration of wheat in 2014.

Growth stage		PCs	Biomass fresh weight (kg ha^-1^)	PCs	Biomass Dry weight (kg ha^-1^)	PCs	*N* uptake (kg ha^-1^)	PCs	*N* concentration (%)
BBCH 19	*R*^2^ cal	4	0.65^∗∗∗^	2	0.53^∗∗∗^	4	0.67^∗∗∗^	5	0.40^∗∗∗^
	*R*^2^ val		0.62^∗∗∗^		0.51^∗∗∗^		0.63^∗∗∗^		0.21^∗∗∗^
	RMSEC		86.4		26		0.8		0.46
	RMSEV		94.3		26.9		0.8		0.53
	Slope cal		0.65		0.53		0.67		0.40
	Slope val		0.63		0.52		0.64		0.30
BBCH 22	*R*^2^ cal	5	0.81^∗∗∗^	2	0.61^∗∗∗^	3	0.80^∗∗∗^	6	0.64^∗∗∗^
	*R*^2^ val		0.76^∗∗∗^		0.58^∗∗∗^		0.77^∗∗∗^		0.54^∗∗∗^
	RMSEC		102.5		46.2		0.8		0.23
	RMSEV		117.2		48.6		0.9		0.29
	Slope cal		0.77		0.61		0.80		0.64
	Slope val		0.76		0.59		0.78		0.54
BBCH 25	*R*^2^ cal	4	0.74^∗∗∗^	2	0.60^∗∗∗^	3	0.75^∗∗∗^	6	0.73^∗∗∗^
	*R*^2^ val		0.71^∗∗∗^		0.56^∗∗∗^		0.72^∗∗∗^		0.60^∗∗∗^
	RMSEC		219.6		57.8		1.4		0.22
	RMSEV		233.3		61.4		1.5		0.27
	Slope cal		0.74		0.60		0.75		0.73
	Slope val		0.72		0.57		0.72		0.64
BBCH 30	*R*^2^ cal	6	0.86^∗∗∗^	4	0.83^∗∗∗^	6	0.87^∗∗∗^	5	0.68^∗∗∗^
	*R*^2^ val		0.80^∗∗∗^		0.75^∗∗∗^		0.75^∗∗∗^		0.51^∗∗∗^
	RMSEC		299.3		89.6		1.9		0.23
	RMSEV		367.4		111.2		2.7		0.30
	Slope cal		0.86		0.83		0.88		0.68
	Slope val		0.82		0.77		0.75		0.58
All BBCH’s	*R*^2^ cal	7	0.93^∗∗∗^	6	0.90^∗∗∗^	7	0.88^∗∗∗^	7	0.66^∗∗∗^
	*R*^2^ val		0.92^∗∗∗^		0.88^∗∗∗^		0.84^∗∗∗^		0.62^∗∗∗^
	RMSEC		212		65.0		1.5		0.38
	RMSEV		241.9		72.0		1.7		0.40
	Slope cal		0.93		0.90		0.88		0.66
	Slope val		0.92		0.88		0.85		0.64


*^∗∗∗^ Statistically significant at *P* ≤ 0.001, respectively. PCs, Number of latent variables.*

**Table 10:** Results of calibration (*R*^2^ cal, RMSEC and slope cal), and seven-fold cross-validation (*R*^2^ val, RMSEV and slope val) partial least square regression models of the relationship between spectral reflectance from 400 to 1000 nm and biomass fresh weight, biomass dry weight, nitrogen uptake and nitrogen concentration of wheat in 2016.

Growth stage		PCs	Biomass dry weight (kg ha^-1^)	PCs	*N* uptake (kg ha^-1^)	PCs	*N* concentration (%)
BBCH 19	*R*^2^ cal	1	0.77^∗∗∗^	1	0.73^∗∗∗^	5	0.74^∗∗∗^
	*R*^2^ val		0.75^∗∗∗^		0.71^∗∗∗^		0.50^∗∗∗^
	RMSEC		47.5		1.2		0.14
	RMSEV		51.5		1.3		0.21
	Slope cal		0.77		0.73		0.74
	Slope val		0.75		0.71		0.60
BBCH 22	*R*^2^ cal	4	0.76^∗∗∗^	1	0.50^∗∗∗^	5	0.81^∗∗∗^
	*R*^2^ val		0.65^∗∗∗^		0.46^∗∗∗^		0.37^∗∗∗^
	RMSEC		59		4		0.10
	RMSEV		74.8		4.2		0.19
	Slope cal		0.76		0.50		0.81
	Slope val		0.69		0.47		0.53
All BBCH’s	*R*^2^ cal	4	0.81^∗∗∗^	2	0.86^∗∗∗^	5	0.95^∗∗∗^
	*R*^2^ val		0.77^∗∗∗^		0.85^∗∗∗^		0.93^∗∗∗^
	RMSEC		59.1		3.2		0.2
	RMSEV		66.6		3.4		0.2
	Slope cal		0.81		0.86		0.95
	Slope val		0.79		0.85		0.93


*^∗∗∗^Statistically significant at *P* ≤ 0.001, respectively. PCs, Number of latent variables.*

## Discussion

In this study, the performance of hyperspectral passive reflectance sensing and digital imaging were tested to assess and predict the biomass and nitrogen status under varying levels of nitrogen fertilizer applications and seeding rates at early development stages from BBCH 19 to BBCH 30. The nitrogen concentration decreased from 3.2 to 2% as mean value in 2014; however, the nitrogen concentration increased from 3.1 to 5% in 2016. The dry weight increased from 450.4 to 2206 kg ha^-1^ as mean values with the wheat development through four biomass samplings in 2014 (**Table 3**). These results agree with other reports (Lemaire and Gastal, 1997; Mistele and Schmidhalter, 2008a), who found that the nitrogen concentration of plants is rather high at early growth stages and decreases with plant development up to the stage of senescence. This reduction in nitrogen concentration is interpreted as a dilution effect. The reason for the increasing nitrogen concentrations in 2016 is most likely due to the application of nitrogen fertilizer between the first and the second sampling date. In 2014, the fertilizer application was conducted 3 days before the first sampling date with no fertilizer applications before the other biomass samplings.

The three methods, the green pixel analysis (%), the spectral indices and the PLSR models, presented a better relationship with the biomass fresh and dry weights, the nitrogen concentration and nitrogen uptake at the latter BBCH stages 22, 25, and 30 than at BBCH 19 in 2014 (**Tables 5**, **7**, **9**). Winter wheat at the very early growth stage was affected by the cold winter weather conditions in 2014, which induced a brownish and yellowish coloring of leaves thus influencing the image analysis. At later development stages, the leaves turned to a green color. A contrasting situation was observed in 2016. Due to a mild winter, the plants showed no discolorations and showed a uniform coloration in both seeding rate treatments, which simplified the pixel analysis. Further, plant soil coverage increases at later growth stages and thus affects less the image analysis.

In this study, the varying seeding and fertilization rates increased the variation in biomass, which, at the latest development stages were approaching full plant coverage, and resulted in distinct differences in the nitrogen status at the early growth stages. A consequence of the increased biomass through plant development as measured in the field of view of the digital camera is a stronger saturation effect at the latest growth stage of BBCH 30 investigated in this study which is also reflected in the upper part of the curvilinear relationships compared to linear ones at earlier development stages. The highest biomass dry weight characterized this, and it turned out to be more difficult to estimate the nitrogen status after reaching 80% green pixels (**Figure 1**). These results agree with studies by Paruelo et al. (2000), who found curvilinear relationships between the percentage of green pixels and green grass biomass (*r* = 0.91) and strong saturation effects became apparent close to 90% total green pixels. Lee and Lee (2013) found that the curvilinear relationships of the canopy cover, derived from the color digital camera image analysis, were best related to the measured shoot nitrogen accumulation (*R*^2^ = 0.87), shoot dry weight (*R*^2^ = 0.71) and leaf area index (*R*^2^ = 0.88) at varying nitrogen fertilization levels, and saturation effects became apparent close to 80% of canopy cover. Wang et al. (2013) observed curvilinear relationships of GMR (the difference between the green channel and the red channel) and G/R (greenness intensity divided by the redness intensity) against the above-ground biomass, the nitrogen content and the leaf area index, respectively, for rice cultivars as well.

A positive relationship was obtained between the green pixel percentage and the biomass fresh and dry weight and the nitrogen uptake and a negative one with the nitrogen concentration of wheat (**Figures 1**, **2**), whereas the relationship to the nitrogen concentration tended to be rather positive in 2014 and negative in 2016. This latter finding is in agreement with Jia et al. (2004, 2014), who found that a significant inverse relationship between the greenness intensity with the nitrogen concentration of wheat was found at the booting growth stage. The negative correlation between green pixels and the nitrogen concentration in 2016, however, might be an exception, due to the later fertilizer application compared to the differing fertilizer management in 2014.

Despite the increased biomass until reaching nearly full plant coverage, no saturation effect was observed for the relationship between the spectral index (R_710_ – R_640_)/(R_710_ + R_640_) and the PLSR model, and their relationships were linear. Almost all normalized spectral indices and normalized difference vegetation indices, which are presented in **Tables 7**, **8**, showed curvilinear relationships with the biomass fresh and dry weight and the nitrogen concentration and uptake (data not shown). The correlation matrices from the contour map analysis depicted higher mean coefficients of determination (*R*^2^) for all dual wavelengths combinations in the range of 302–1148 nm (as normalized difference index) of the reflectance sensor with the biomass fresh and dry weight and the nitrogen uptake compared with the nitrogen concentration. **Tables 7**, **8** shows that closer relationships existed between all spectral indices with the biomass weight and dry weights and the nitrogen uptake compared with the nitrogen concentration. This finding is in agreement with other studies. Lukina et al. (1999) showed that the NDVI was strongly related to the dry weight (*r* = 0.71) and nitrogen uptake (*r* = 0.81) compared with the nitrogen concentration (*r* = 0.33) under four nitrogen rates. Similarly, Erdle et al. (2011) reported that the NDVI yielded better relationships with the nitrogen uptake compared with the nitrogen concentration across different growth stages of winter wheat.

The calibration model yielded by the PLSR analysis was more closely related to the nitrogen concentration compared with the spectral indices and green pixel percentage (**Tables 5**–**10**). Comparably, in winter wheat, the prediction of the nitrogen concentration was enhanced when using PLSR models compared to the previously assayed spectral indices (**Tables 7**–**10**). These results are in line with Li et al. (2014), who indicated that PLSR can be a useful approach to derive canopy nitrogen concentration of winter wheat across growth stages, cultivars, sites and years under field conditions in comparison to spectral indices. The advantage of PLSR models compared with the green pixel analysis and spectral index models is that the PLSR uses information from spectral bands from 400 to 1000 nm and selects the number of factors to best represent the calibration data avoiding overfitting. PLSR did not indicate limitations in predicting the nitrogen uptake and biomass fresh and dry weight and the nitrogen uptake (**Figures 5**, **6**), and the relationships between the observed and predicted values were linear. On the other hand, relationships obtained from the development of simple spectral reflectance indices or PLSR models did not substantially differ. Several indices, newly developed and existing ones, proved to deliver consistent and strong relationships to the biomass fresh and dry weight and the nitrogen uptake at BBCH stages 22, 25, and 30, requiring no specific growth-stage specific models. This makes them somehow advantageous compared to PLSR models requiring further testing in other environments to test their robustness. Simple indices can also more easily be transferred to simpler sensors easier to handle and being less costly than hyperspectral sensors.

## Conclusion

In conclusion, the results show that the models developed from the green pixel analysis, spectral indices analysis and PLSR analysis allowed to assess with varying quality the biomass fresh weight and biomass dry weight and the nitrogen uptake at early development stages. The digital image analysis showed a stronger saturation effect at growth stage BBCH 30, the nitrogen status of which proved to be the most difficult to estimate after reaching 80% green pixel coverage. PLSR models potentially improve the assessment of the nitrogen concentration of winter wheat compared with spectral indices and the green pixels analysis. The results indicate that the non-destructive assessment of the biomass and nitrogen status of wheat is possible by means of spectral sensing at early crop growth stages. Methods established under highly controlled experimental conditions could successfully be verified under farmer’s field conditions. A subsequent development and testing of fertilization algorithms will have to be done as well. It will be interesting to leverage the potential from ground-based imaging to UAV-based sensing. The results so far indicate that reflectance-based spectral sensing allows to detect differences in the biomass and nitrogen status already at very early growth stages in the tillering phase. A further improvement at the very earliest tillering stage might further be obtained by choosing an oblique view of angle which augments the biomass seen. However, at later growth stages this is not to be recommended once an increased leaf area index is achieved. Taken together, this might pave the way for more informed management decisions and potentially lead to improved nitrogen fertilizer management at early development stages.

## Author Contributions

US, SE, and GB conceived and designed the experiments. SE and GB performed the experiments and analyzed the data. SE, GB, and US wrote the paper.

## Conflict of Interest Statement

The authors declare that the research was conducted in the absence of any commercial or financial relationships that could be construed as a potential conflict of interest.
