# Corrigendum: Passive Reflectance Sensing and Digital Image Analysis Allows for Assessing the Biomass and Nitrogen Status of Wheat in Early and Late Tillering Stages

**DOI:** 10.3389/fpls.2021.670027

**Published:** 2021-04-20

**Authors:** Salah Elsayed, Gero Barmeier, Urs Schmidhalter

**Affiliations:** ^1^Evaluation of Natural Resources Department, Environmental Studies and Research Institute, University of Sadat City, Sadat, Egypt; ^2^Department of Plant Sciences, Technical University of Munich, Freising, Germany

**Keywords:** digital agriculture, high-throughput sensing, imaging, nitrogen management, phenomics, precision phenotyping, precision farming, spectrometry

In the original published article, the affiliation of the author Salah Elsayed was incorrectly displayed as “Department of Plant Sciences, Technical University of Munich, Freising, Germany.”

The correct affiliation for this author which was incorrectly displayed as this author's present address in the original article, should have been:

“Evaluation of Natural Resources Department, Environmental Studies and Research Institute, University of Sadat City, Sadat, Egypt”

The authors apologize for this error and state that this does not change the scientific conclusions of the article in any way. The original article has been updated.

